# Different host factors are associated with patterns in bacterial and fungal gut microbiota in Slovenian healthy cohort

**DOI:** 10.1371/journal.pone.0209209

**Published:** 2018-12-20

**Authors:** Aleksander Mahnic, Maja Rupnik

**Affiliations:** 1 Department for Microbiological Research, National Laboratory for Health, Environment and Food, Maribor, Slovenia; 2 Faculty of Medicine, University of Maribor, Maribor, Slovenia; University of Illinois at Chicago College of Medicine, UNITED STATES

## Abstract

Gut microbiota in a healthy population is shaped by various geographic, demographic and lifestyle factors. Although the majority of research remains focused on the bacterial community, recent efforts to include the remaining microbial members like viruses, archaea and especially fungi revealed various functions they perform in the gut. Using the amplicon sequencing approach we analysed bacterial and fungal gut communities in a Slovenian cohort of 186 healthy volunteers. In the bacterial microbiome we detected 253 different genera. A core microbiome analysis revealed high consistency with previous studies, most prominently showing that genera *Faecalibacterium*, *Bacteroides* and *Roseburia* regularly comprise the core community. We detected a total of 195 fungal genera, but the majority of these showed low prevalence and are likely transient foodborne contaminations. The fungal community showed a low diversity per sample and a large interindividual variability. The most abundant fungi were *Saccharomyces cerevisiae* and *Candida albicans*. These, along with representatives from genera *Penicillium* and *Debaryomyces*, cover 82% of obtained reads. We report three significant questionnaire-based host covariates associated with microbiota composition. Bacterial community was associated with age and gender. More specifically, bacterial diversity was increased with age and was higher in the female population compared to male. The analysis of fungal community showed that more time dedicated to physical activity resulted in a higher fungal diversity and lower abundance of *S*. *cerevisiae*. This is likely dependent on different diets, which were reported by participants according to the respective rates of physical activity.

## Introduction

The human gut microbiota is a diverse community comprised of bacteria, fungi, archaea, viruses and protozoa. These microorganisms co-exist in a complex interdependence, shaped by countless microbe-microbe and microbe-host interactions. Bacteria dominate the gut microbiome, representing approximately 99.9% of the total cell population. Fungi, archaea and protozoa combine to fulfill the remaining 0.1% [1,2]. Although bacterial community remains the primary focus of gut microbiota research, recent studies on archaea [3,4], viruses [5,6] and fungi (mycobiome) [7–9] indicate potential involvement of these microbial groups in health and disease.

Studies on bacterial microbiota largely focus on the comparison between different patient populations and healthy controls, striving to define disease specific microbial patterns [10–12]. Major steps towards understanding the healthy microbiota were made in 2008 when both the American Human Microbiome Project (NIH) and the European MetaHIT project launched with the objective to optimize and standardize analytical methods, and increase the size of studied cohorts in order to better address the interindividual variability [1,13,14]. Human gut microbiota was, up until now, mostly studied in relation to age, diet and lifestyle-related changes [2,15–19] as well as human genetics by screening twin-pairs [20]. Noteworthy are two recent large cohort studies on healthy Belgian and Dutch populations, which succeeded to associate bacterial microbiome patterns with a comprehensive collection of host and environmental factors [21,22].

The fungal community received little attention up to date, especially in the healthy population. Reported concentrations of fungi in stool samples range from 0 to 10^9^ CFU g^-1^ per stool, indicating high interindividual variability [23,24], but the discrepancies between culture-dependent and culture-independent methods question the reliability of these estimates [23,25,26]. Even at several magnitudes lower count compared to the bacteria, fungi show significant patterns in different gastrointestinal and other diseases, especially in the immune-compromised patient populations [27], as well as various interactions with the host immune system [28]. The beneficial effects of fungi in human gastrointestinal tract, on the other hand, are not well known. Certain commercially available probiotics already utilize the ability of *Saccharomyces* strains to limit the inflammatory response and increase immune health [29]. Certain filamentous fungi with the potential to metabolize complex plant-derived carbohydrates were primarily studied in insects [30] and rumen of cattle [31], but have also been detected in humans [8].

Here we present a study including a Slovenian cohort of 186 healthy volunteers. Both the bacterial and fungal gut community structures were analyzed in relation to a set of 13 host specific factors. We report age- and gender-associated patterns in the bacterial communities, and a weak association between the time dedicated to physical activity and the fungal community, which is likely influenced by different dietary habits coinciding with physical activity.

## Methods

Stool samples were collected from 186 healthy volunteers from Maribor (Slovenia) and the surrounding area. Samples included in the final analysis were required to be from participants who were at least 18 years old and without any gastrointestinal infection or surgical procedure on the gastrointestinal tract 3 months prior to sample collection. Participants diagnosed with chronic inflammatory diseases were also excluded. Stool samples were collected together with a completed questionnaire and a written informed consent in accordance with the approval of the Republic of Slovenia National Medical Ethic Committee. Upon collection, each sample was anonymized, deidentified and was further processed only with a study code.

Questionnaire covered information on volunteers age, gender, body mass index, type of diet (regular, vegetarian, vegan, lactose free, gluten free or raw food), antibiotic therapy in last 3 months (yes or no), hospitalization in last 3 months (yes or no), digestion (regular, occasional constipation or regular constipation), physical activity (none or occasional exercise, exercise approximately once a week, exercise multiple times weekly or active athlete), surgical removal of cecum (yes or no), probiotics or prebiotics usage (yes or no), smoking (yes or no) and level of stress (1–5) (Table 1).

**Table 1:** Host/environmental factors with sample distribution and PERMANOVA analysis. A list of host and environmental factors, which were collected with questionnaire, with corresponding distribution of samples inside their respective factor category. To the right we show the results of the PERMANOVA test (1000 permutations, Bray-Curtis distances), presented with the value for explained variance (R^2^) for each factor in relation to the bacterial or fungal community. Host/environmental factors with a significant *P* value, after adjustment with Benjamini-Hochberg correction (FDR < 0.05), are highlighted in grey.

			PERMANOVA	
Host factor	Categories	Distribution of samples	Explained variance (R^2^) in bacterial community	Explained variance (R^2^) in fungal community
Gender	Male	69 (37.1%)	**0.01114** **(*P* = 0.026)**	0.00446
	Female	117 (62.9%)		
Age		min 18, max 85, mean 45.2	**0.01065** **(*P* = 0.045)**	0.00387
Diet	Regular	151 (81.2%)	0.03633	0.05478
	Vegetarian	23 (12.4%)		
	Vegan	5 (2.7%)		
	Gluten free	2 (1.1%)		
	Lactose free	4 (2.2%)		
	Raw food diet	1 (0.5%)		
BMI		min 16.5, max 38.7, mean 24.8	0.0081	0.00622
Antibiotic therapy in the last 3 months	Yes	12 (6.5%)	0.00724	0.00519
	No	174 (93.5%)		
Hospitalization in the last 3 months	Yes	5 (2.7%)	0.0063	0.00333
	No	181 (97.3%)		
Digestion rate	Regular	158 (84.9%)	0.00598	0.00272
	Occasional constipation	25 (13.4%)		
	Frequent constipation	3 (1.6%)		
Time dedicated to physical activity	Occasionally	75 (40.3%)	0.00578	**0.04188** **(*P* = 0.026)**
	Once per week	49 (26.3%)		
	Multiple times per week	58 (31.2%)		
	Active athlete	4 (2.2%)		
Cecum removal	Yes	11 (5.9%)	0.00579	0.00238
	No	175 (94.1%)		
Chronic disease	None	166 (89.2%)	0.01654	0.01203
	IBS	3 (1.6%)		
	Unspecific	17 (9.1%)		
Probiotics or prebiotics usage in the last 3 months	Yes	27 (14.5%)	0.00441	0.00744
	No	159 (85.5%)		
Smoker	Yes	20 (10.8%)	0.00439	0.00433
	No	166 (89.2%)		
Stress	1–5	mean 3.151	0.00389	0.03556

Stool samples were collected in sterile containers. Faecal material was mixed thoroughly with an inoculation loop, a portion corresponding to approximately 50 μL was stored in 1 mL of Inhibitex buffer (QIAamp Fast Stool DNA Mini Kit, Qiagen) and frozen at -80°C until further use.

### Isolation of the total bacterial DNA and high-throughput 16S rDNA amplicon sequencing

Total DNA was isolated from stool samples using QIAamp Fast Stool DNA Mini Kit (Qiagen, Hilden, Germany) with mechanical disruption (MagNA Lyser, speed 7000 for 70 s).

The bacterial community composition was determined by sequencing the V3V4 hypervariable region of the 16S rRNA gene using a broad-range set of primers Bakt_341F (5'-CCTACGGGNGGCWGCAG-3')–Bakt_805R (5'-GACTACHVGGGTATCTAATCC-3') [32]. The library preparation was performed according to the recommended Illumina 16S Metagenomic Sequencing Library Preparation manual protocol (Illumina, CA, USA). The fungal community composition was determined by sequencing the Internal Transcribed Spacer 2 (ITS2) using a broad-range set of primers ITS86F (5'-GTGAATCATCGAATCTTTGAA-3')–ITS4R (5'-TCCTCCGCTTATTGATATGC-3') [33]. The library was prepared according to the 16S Metagenomic Sequencing Library Preparation manual (Illumina, CA, USA) with the exception of using Q5 High-Fidelity DNA Polymerase (NEB, Massachusetts, USA) instead of the recommended KAPA HiFi HotStart ReadyMix (Kapa Biosystems, Massachusetts, USA). Sequencing was performed on the Illumina MiSeq platform with MiSeq Reagent Kit V3 (2x300 cycle, 10% PhiX).

### Sequence data analysis

The analysis in mothur (v.1.36.1) [34,35] was done according to the MiSeq standard operating procedure (SOP) for Illumina paired end reads. The bacterial 16S rRNA reads were processed using the following criteria: i) reads were not allowed any ambiguous bases and the maximum homopolymer length was set to 8 base pairs (bp); ii) The reads were aligned against the Silva reference alignment (Release 123); iii) Chimeras were identified using the UCHIME algorithm; iv) The classification of reads was performed using the RDP training set (v.9) with 0.80 bootstrap threshold value; v) Sequences were clustered into operational taxonomic units (OTUs) at the 97% similarity cut-off. After quality filtering we obtained an average depth of 35484 reads per sample (min 297, max 89229). OTUs represented in the abundance less than 0.01% of total number of reads were removed followed by rarefying each sample to 3000 reads. Samples with less than 3000 reads were removed from further analysis (n = 1). Alternatively, normalization of contingency table as described by Lagkouvardos et al., 2017 [36] was tested, but did not significantly impact the final set of OTUs nor their relative abundance. Method reportedly introduces lower bias in the representation of low abundant OTUs, but this advantage was not observable in our dataset due to our conservative approach to remove OTUs with overall abundance less than 0.01%.

Fungal ITS2 reads were processed using following criteria: i) The reads were not allowed any ambiguous bases; ii) The removal of reads shorter than 205 bp or longer than 502 bp; iii) The removal of reads containing homopolymers longer than 12 bp; iv) ITSx software was used for binning in order to remove non-fungal reads; v) The reads were aligned pairwise using the Needleman-Wunsch method (rewards +1 for a match and penalizes with -1 and -2 for a mismatch and gap, respectively); vi) The sequences were clustered into operational taxonomic units (OTUs) at a 98% similarity cut-off; vii) The classification was inferred using UNITE ITS database (version 6) with 0.80 bootstrap threshold value. After quality filtering we obtained an average depth of 20901 reads per sample (min 504, max 66742). OTUs represented in the abundance less than 0.01% of total number of reads were removed followed by rarefying each sample to 950 reads. Samples with less than 950 reads were removed from further analysis (n = 6).

The sequence data was deposited in the form of combined paired end reads (contigs) on the Metagenomics RAST (MG-RAST) database server (http://metagenomics.anl.gov/) under the project access number mgp85661 (https://www.mg-rast.org/linkin.cgi?project=mgp85661). Seven samples (all fungal ITS2 metagenomes) did not meet the minimum criteria of 1000000 bp per sample as required by MG-RAST and are available along with metadata for all samples in Supporting Information (S1 Appendix).

The statistics and graphic representation were done in mothur (v 1.31.1) and R (version 3.1.3) using packages ‘ggplot2’ and ‘vegan’.

### Core community analysis

The core community was defined in our study as selection of OTUs with a relative abundance of at least 0.1% and present in more than 95% of tested samples. To compare our results with already published data, we found 4 studies, in which comparable information was either reported in the article or could be extracted from supplementary information. These studies include 1) a study on combined Belgian Flemish Gut Flora Project (FGFP; discovery cohort; N = 1106) and the Dutch LifeLines-DEEP study (LLDeep; replication; N = 1135); The criteria for a core community was the presence of a genus in at least 95% of the tested population [21]; 2) A study on the collection of samples from the Human Microbiome Project (HMP, n = 238); The criteria for a core community was the presence of OTUs in at least 95% of the tested population [37]; 3) A study on a Mongolian cohort (n = 64); The criteria for a core community was the presence of OTUs in at least 90% of the tested population [38] and 4) a study on European individuals (n = 124). Authors used shotgun metagenomic sequencing, therefore the results were presented at the species taxonomic level. To ensure consistency, we used only information on genus taxonomy with the criteria of taxa being detected at the minimum 10% reference coverage and present in at least 95% of the tested samples [1].

## Results and discussion

### Analysis of bacterial community

**Fig 1:**
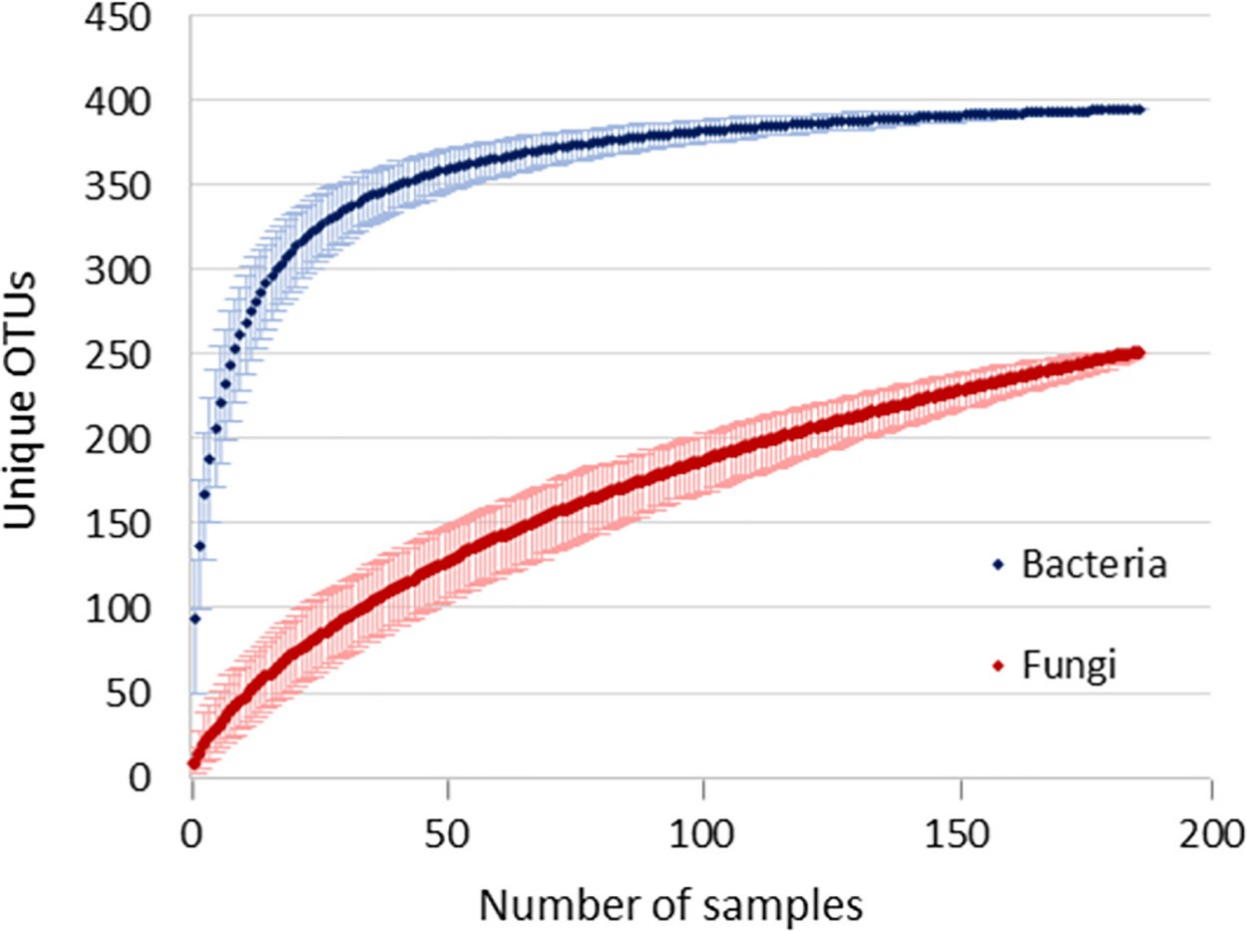
Rarefaction curves for bacterial and fungal OTUs. Rarefaction curves for bacterial (blue) and fungal (red) OTUs were calculated by rarefying both bacterial and fungal community to 950 reads/sample (only for this specific analysis we rarefied bacterial community to 950 reads/sample in order for the rarefaction curves to be comparable). Plotted data points represent the mean value of OTUs for the respective number of samples (1000 iterations) with a 95% confidence interval.

**Fig 2:**
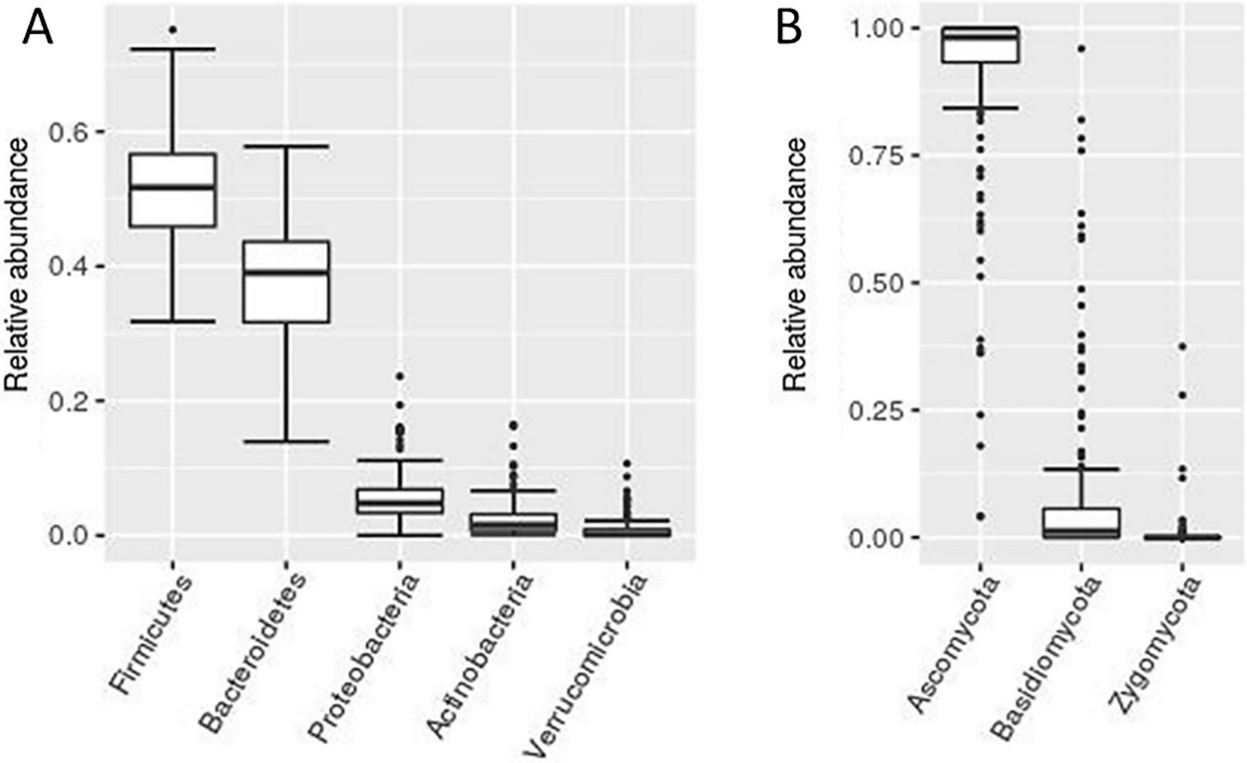
Bacterial and fungal phyla relative abundance. A box plot presentation of relative abundances of bacterial (A) and fungal (B) phyla. Only phyla with an overall relative abundance greater than 1% are shown.

Amplicon sequencing approach targeting V3V4 variable region of 16S rRNA gene was used to investigate the bacterial communities in a group of 186 healthy volunteers from the Slovenian population. After quality filtering we obtained an overall richness of 27852 OTUs corresponding to 253 bacterial genera. This falls short off the projected richness of 294 genera (Chao1 richness index), which would require an estimated 702 additional samples to reach. After the removal of low abundance OTUs (overall relative abundance < 0.01%) and subsampling the remainder to 3000 reads per sample, we obtained 395 bacterial OTUs (Fig 1), on average 121.2 OTUs per sample (S1 Table). Out of 7 detected bacterial phyla, *Firmicutes* and *Bacteroidetes* show the highest abundance as well as interindividual variability (Fig 2A), but in contrast to some related studies, we found no significant correlation between the *Firmicutes*/*Bacteroidetes* ratio and host/environmental factors [39,40].

**Fig 3:**
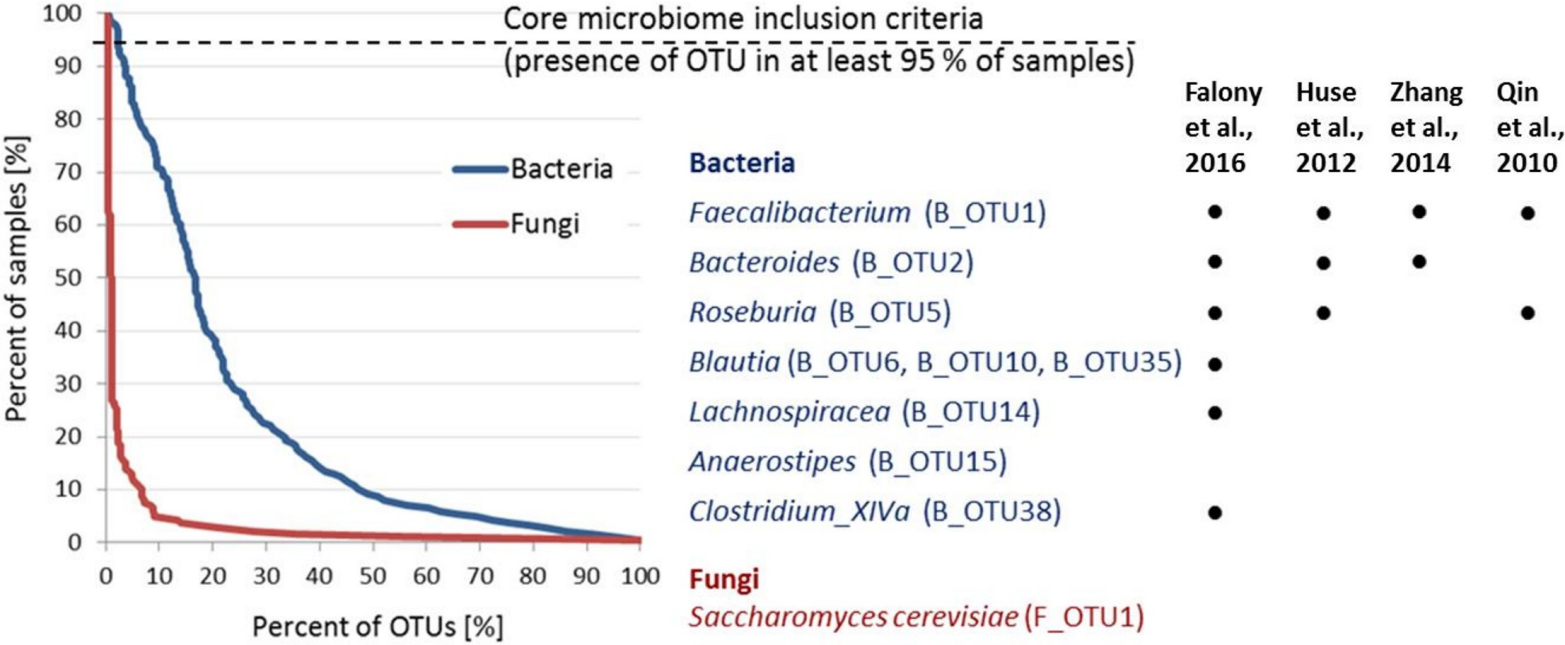
Core microbiome analysis. The core microbiome is shown as a percent of samples (%) that include the corresponding percent (%) of fungal (red) and bacterial (blue) OTUs. To the right is the list of bacterial (n = 9) and fungal (n = 1) OTUs, which meet the criteria for inclusion into the core community. We compared our observed core taxa with four other studies also reporting core communities (information on studied cohorts and core community inclusion criteria are included in Materials and Methods). The dot indicates that the core taxa identified in our cohort was also reported by respective study at genus or family taxonomic level.

The bacterial core community, defined as OTUs with relative abundance of at least 0.1% and present in 95% of samples or more, consists of 9 OTUs, classified into 7 different bacterial genera (Fig 3). The two most abundant core OTUs correspond to the genus *Faecalibacterium* (B_OTU1; B_ and F_ prefixes before OTU indicate bacterial and fungal OTU, respectively) and *Bacteroides* (B_OTU2). The remaining 7 OTUs all classify to the family *Lachnospiraceae*, with the most prevalent being genus *Blautia* (B_OTU6, B_OTU10, B_OTU35) along with single representatives from genera *Roseburia* (B_OTU5), *Lachnospiracea* (B_OTU14), *Anaerostipes* (B_OTU15) and *Clostridium XIVa* (B_OTU38) (Fig 3, S1 Table).

The bacterial core microbiome in healthy population was previously analyzed in different studies, including European [1,21], American [37] and Mongolian cohorts [38]. Despite the different methodologies used, we observed a high degree of consensus with their results, especially in the case of the combined dataset from FGFP (Belgian Flemish healthy cohort), LLDeep (Dutch healthy cohort) and some other U.K. and U.S. studies [21] (Fig 3). Among the 14 core genera reported in this combined dataset, 6 coincide with our set of core OTUs, although only at the family taxonomic level in case of *Lachnospiraceae*. Genus *Anaerostipes* was the only additionally detected genus in our analysis while 7 genera were detected by Falony et al., but not by us [21]. Comparing our results to all four other studies we found that at this core inclusion criteria, *Faecalibacterium* always comprises core community, while *Bacteroides* and *Roseburia* each failed to be included into core community by only one of the other four studies. It should be noted that in the study by Qin et al., *Bacteroides vulgatus* missed the core community inclusion criteria by a single study participant. Other core genera reported by four comparator studies vary substantially among investigated cohorts [21,37] (Fig 3). Recently, researchers became more inclined to investigate the bacterial “functional core” using shotgun metagenomic approach [41]. Functional profile showed more redundancy among individuals [42] as a result of same metabolic traits being performed by a variety of different bacterial groups. Still, our findings in congregation with others support, to some extent, the “outdated” idea of a taxonomical core. Observed core genera either indicate a common microbial evolution [43] or a possession of a unique set of traits which facilitate their persistence in the gut despite the alternating environment. Defining the core community at the species level and identifying unique metabolic traits of these taxa might further elucidate this observations.

### Age and gender associated differences in bacterial community

We identified gender and age to be significantly associated with the bacterial microbiome, jointly explaining 2.2% of the interindividual bacterial community variation (Permutational multivariate analysis of variance (PERMANOVA) using Bray-Curtis distances, false discovery rate (FDR) < 0.05). All host/environmental factors, collected with the questionnaire, and PERMANOVA test results are shown in Table 1.

**Fig 4:**
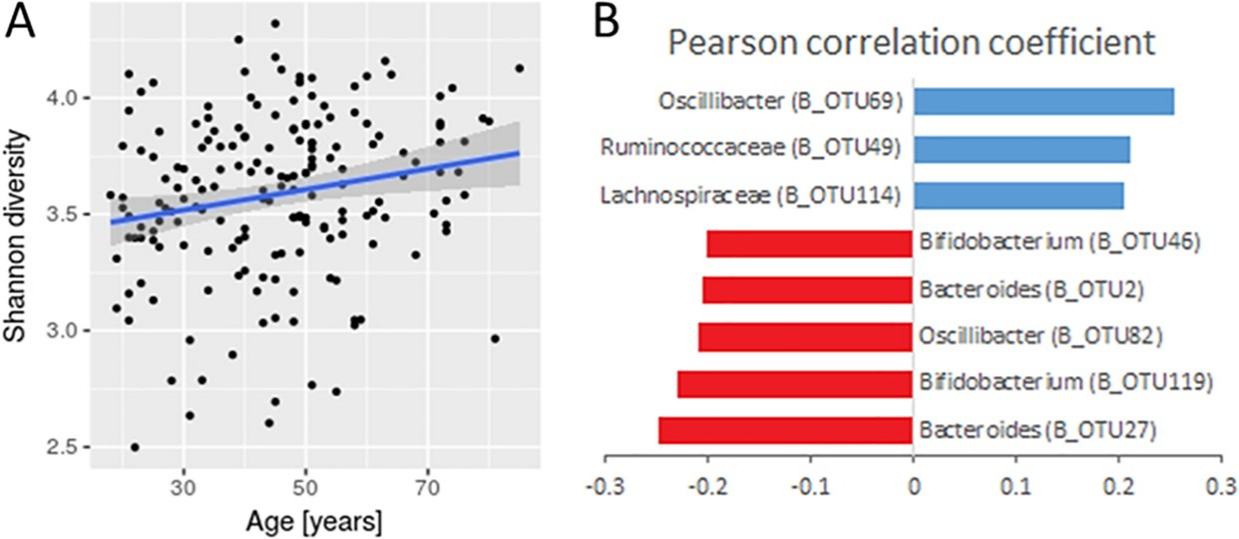
Age-associated changes in bacterial communities. Graph shows bacterial community Shannon diversity index in relation to age. The linear regression indicates the increase of the Shannon diversity with age and is presented with a 95% confidence interval (A) (Pearson's r = 0.213, P = 0.003). The bar plot shows Pearson correlations of bacterial OTUs that significantly increase (blue) or decrease (red) in relative abundance with age (FDR < 0.05) (B).

We observed an age-associated increase in bacterial richness and diversity (Pearson's r = 0.217, P = 0.003 and r = 0.213, P = 0.003 for Chao1 and Shannon indices, respectively) (Fig 4A). The Pearson correlation test most prominently showed an age-related decrease in the genera *Bifidobacterium* (B_OTU46, B_OTU119) and *Bacteroides* (B_OTU2, B_OTU27), and an increase in representatives from the genus *Clostridiales* (B_OTU49, B_OTU69, B_OTU114) and unclassified *Proteobacteria* (B_OTU93) (Fig 4B).

Multiple studies have looked into the dynamics of microbiota throughout the human lifespan with mostly contradicting results. As previously reported, we also observed an age-related increase in *Proteobacteria* and a decrease in the genera *Bifidoacterium* and *Bacteroides* [44–46]

However, we did not confirm the often-reported age-associated decrease of *Faecalibacterium* and *Clostridium cluster XI* [47,48]. In addition, a significant increase in known short chain fatty acids producers from families *Ruminococacceae* and *Lachnospiraceae* observed in this study to some extent contradict the previously reported lower availability of total SCFAs in the elderly population [19]. The decline in SCFAs levels and other changes in metabolome were suggested to be linked with a transition from a saccharolytic metabolism, typical in adults, towards a predominantly putrefactive metabolism [49]. But these changes might very well be environment dependent and therefore vary between cohorts based on subjects long-term and short-term dietary habits [50].

Contrary to common narrative [47,49], we observed a slight increase in bacterial community diversity and richness with age, but similar trends have already been reported by others [22,51]. The discrepancies between studies likely arise from generation-specific dietary habits and lifestyle. Therefore, longitudinal studies, accounting for interindividual variability and age-related physiological changes, are needed to improve our knowledge on aging microbiota.

**Fig 5:**
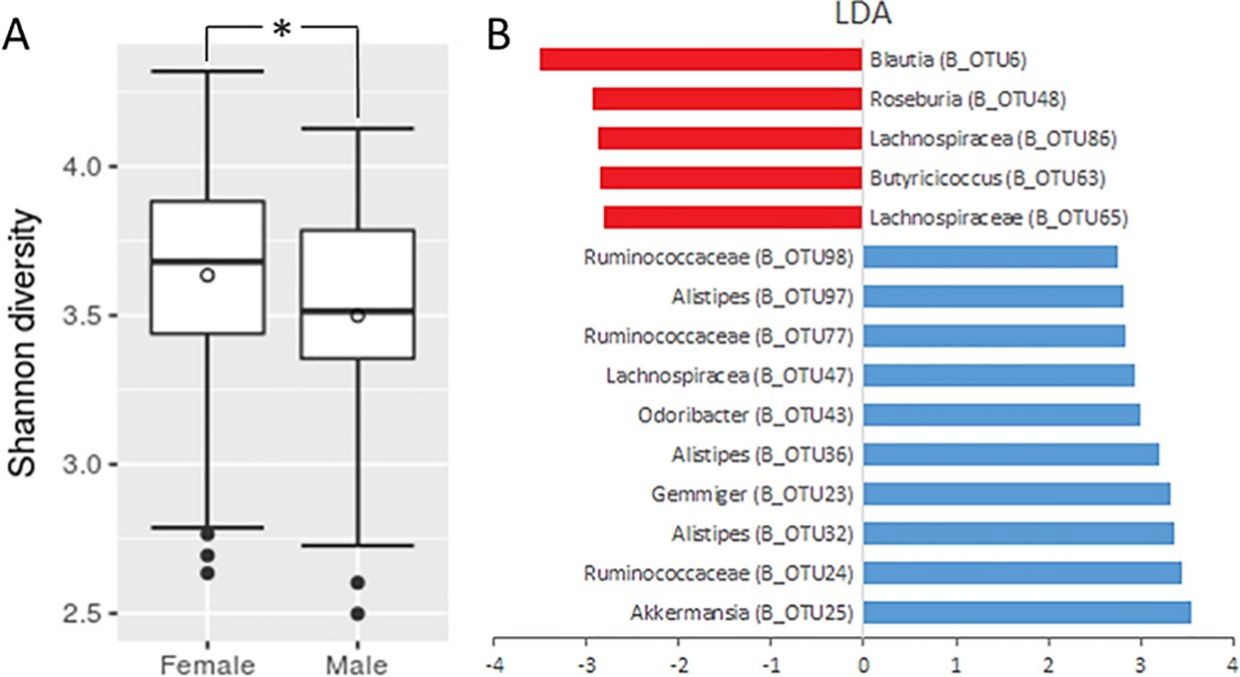
Gender-associated differences in bacterial communities. A box plot presentation of Shannon diversity indices in females compared to males (Kruskal-Wallis test, *P* = 0.014) (A). The bar plot presents LEfSe results showing LDA values for OTUs, which were significantly increased in males (red) and females (blue).

Bacterial communities in our cohort significantly differ between males and females (AMOVA, P < 0.001), with females showing a slightly higher Shannon diversity (Kruskal-Wallis test, *P* = 0.014) (Fig 3A) (Fig 5A). In the male population, we observed a significant increase in the abundance of several OTUs from the order *Clostridiales* (B_OTU6, B_OTU48, B_OTU63, B_OTU65, B_OTU86), while females most prominently display higher abundance of *Akkermansia* (B_OTU25) in addition to multiple OTUs corresponding to the family *Ruminococcaceae* (B_OTU24, B_OTU77, B_OTU98) and genus *Alistipes* (B_OTU32, B_OTU34, B_OTU97) (Fig 5B).

Gender often ranks high among host covariates associated with bacterial microbiota [21,22,52,53]. In agreement with our findings, Borgo et al. and Zhernakova et al. already reported higher bacterial diversity in female populations. It should be noted that Borgo et al. reported gender specific changes in bacterial diversity exclusively for mucosa-associated microbiota, while lumen-associated microbiota showed no alpha or beta diversity distinction between genders [22,53]. Specific gender-associated patterns in bacterial community vary among studies. Our observed changes are predominantly characterized by a rearrangement of several taxa corresponding to *Firmicutes*, while Dominianni et al. most notably showed a female-associated decrease in *Bacteroidetes* and Borg et al. reports a female mucosa-associated enrichment with *Bifidobacterium* and a depletion in *Veillonellaceae* [52,53]. These patterns do not seem to be diet-related, since dietary groups in our cohort showed a balanced distribution between genders. Additionally, both host covariates (gender and diet) showed non-related changes in bacterial communities (nested PERMANOVA, *P* = 0.370). Nevertheless, it should be noted, that energy metabolism varies between genders even under the same dietary regime [54,55]. In agreement with previous studies, we conclude that gut microbiota shows gender-specific patterns, but these seem to be cohort specific. Better understanding will follow from an incorporation of gender-associated differences in physiology [56] and immune response [57] into gut microbiota studies.

### Analysis of fungal communities

Amplicon sequencing of ITS2 spacer region was used to investigate fungal communities. After quality filtering we obtained an overall richness of 2158 OTUs corresponding to 195 fungal genera, which is just one short of predicted 196 (Chao1 richness index), indicating that the majority of the present fungal genera were most likely detected. After the removal of low abundance OTUs (overall relative abundance < 0.01%) and rarefying the remainder to 950 reads per sample we obtained 251 fungal OTUs (Fig 1), on average 7.5 OTUs per sample (S2 Table). Analysed fungal community is largely dominated by representatives from *Ascomycota* (Fig 2B), further emphasized by the fact that the 4 most abundant fungal OTUs, *Saccharomyces cerevisiae* (F_OTU1), *Candida albicans* (F_OTU2) and unclassified species from genera *Penicillium* (F_OTU3) and *Debaryomyces* (F_OTU4) (detected in 98.9%, 61.8%, 21% and 50% of samples, respectively), together cover over 80% of total obtained reads. In comparison, it requires top 82 bacterial OTUs to reach the same total read coverage. Fungal communities exhibit low diversity and high interindividual variability. Individual fungal OTUs appear on average in 5.6 out of 186 tested samples (3%). Consequently, only *Saccharomyces cerevisiae* met the criteria for inclusion in the core community (Fig 3, S2 Table). However, it is highly likely that substantial proportion of *S*. *cerevisiae* sequences were food derived.

Compared to bacteria, fungi introduce the additional problem of differentiating between gut commensals and transient colonizers, which derive primarily from food. Generally, the minimum criteria to consider a fungus as a potential commensal, is its successful growth at 37°C [58]. Although multiple species meet this criteria, some studies rightfully question the ability of fungi to persist in the gut microbiota [9]. Still, it is important to note that mucosa-associated fungi reportedly show more stability compared to luminal communities [59]. Yeast *S*. *cerevisiae* is usually among the most abundant fungi detected in the gut [9,60] and is also the most prevalent as well as abundant fungal OTU in our study. It was shown though, that despite the ability to grow at 37°C, *S*. *cerevisiae* does not persist in the gut for more than about 3 days [61], and is therefore not considered a true commensal. Fungi most often reported to colonize the gut include representatives from the genera *Candida*, *Malassezia*, *Cladosporium* and yeast from the *Dipodascaceae* family [8,62]. Other commonly detected fungi in the gut, which are not able to grow at 37°C, include foodborne species from the genera *Debaryomyces* and *Penicillium*. These fungi are often used at different food processing stages or are present as unwanted food contaminants. They can also comprise normal skin or oral microbiota [15,63–66]. A recent publication investigated the composition changes of fungal communities during periods of controlled diet [9]. The authors demonstrated the importance of food and the oral cavity as the major sources of commonly detected fungi, showing that switching to a *S*. *cerevisiae* free diet or improving oral hygiene resulted in a significant decrease of *S*. *cerevisiae* or *C*. *albicans* abundance in stool, respectively [9].

### Differences in fungal communities associated with physical activity

**Fig 6:**
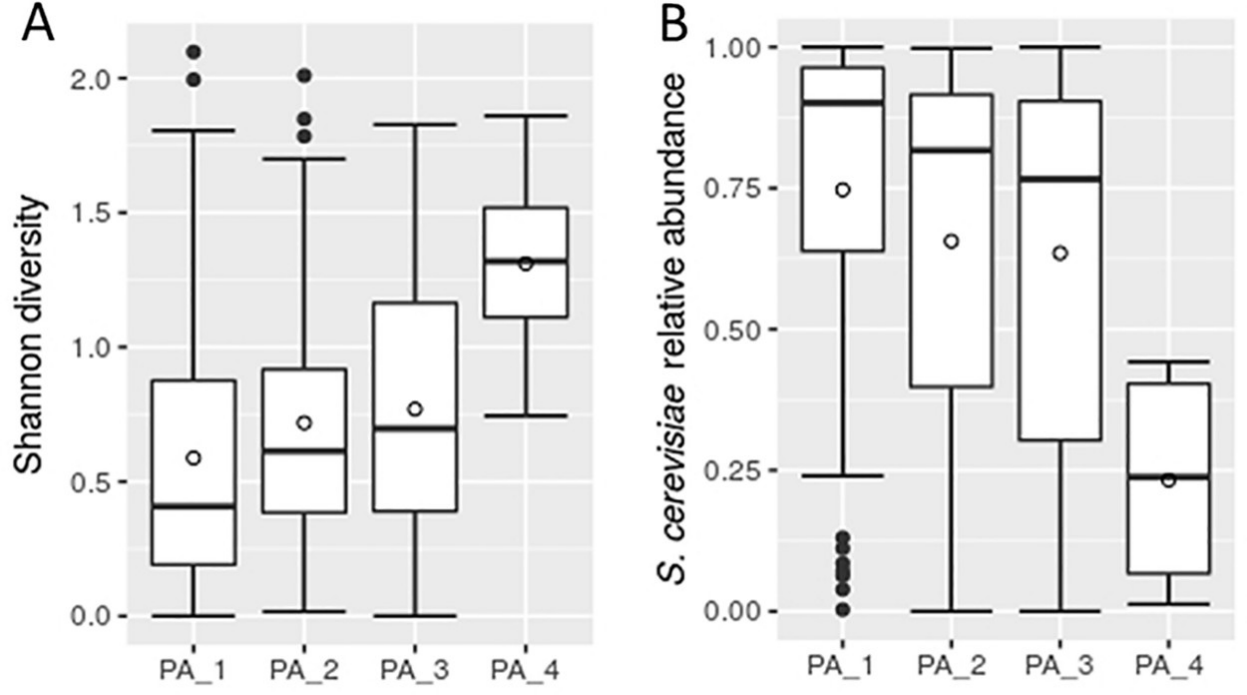
Changes in fungal microbiota associated with the reported rate of physical activity. Box plots presenting the Shannon diversity indices (A) and *S*. *cerevisiae* abundance (B) according to the rate of reported physical activity. Physical activity was defined with four categories, i.e. PA_1—occasional exercise; PA_2—exercise approximately once a week; PA_3—exercise multiple times weekly; PA_4—active athlete.

Physical activity was the only significant covariate associated with fungal microbiome, explaining 4.2% of interindividual fungal community variation (PERMANOVA using Bray-Curtis distances, false discovery rate (FDR) < 0.05). The physical activity factor was defined in the questionnaire with 4 categories depending on the time participants dedicated to sport and recreational activities, ranging from none or occasional exercise to an active athlete. We found, that more frequent physical activity correlated with an increase in the total fungal diversity (Spearman's r = 0.217, P = 0.003) and a decrease in the abundance of *S*. *cerevisiae* (F_OTU1) (Spearman's r = -0.217, P = 0.003) (Fig 6). Additionally we report, that *S*. *cerevisiae* was significantly associated with lower overall fungal community diversity (Pearson's r = 0.712, P<0.001) (S1 Fig), further supported by negative correlations it exhibits with highly abundant *C*. *albicans* (F_OTU2) and unclassified *Debaryomyces* (F_OTU4) (S3 Fig).

Distinct patterns, associated with physical activity, were recently reported in relation to bacterial communities. Authors mainly emphasized the physical activity-associated increase in bacterial diversity and the decrease in relative abundance of *Bacteroides* [67,68], but we did not observe any related changes in the bacterial community analysed in our cohort. We would like to note, that the physical activity variable showed correlation with different dietary habits these groups reported. Most notably, there were significant discrepancies in the proportion of participants with diet exempting or reducing meat consumption (veganism and vegetarianism). The proportion of participants reporting veganism/vegetarianism was 8.7%, 8.9%, 29.3% and 50% for the 4 groups ascending by the time dedicated to physical activity. Diet was also the second most prominent covariate in the PERMANOVA test (explained variance (R2) of 5.5%, *P* = 0.007) (Table 1), but did not manage to meet the false discovery rate (FDR) significance threshold.

The specific nature of fungal community compositions, especially the low richness and the high interindividual variability, makes it challenging to determine significant correlations with host factors. Conclusions from related studies confirm this observation, as Nash et al. were unable to correlate any host factors with fungal community, and Auchtung et al. reported high temporal variability, mainly attributed to short term diet [8,9]. To our knowledge, the only other group that reported fungal community associated host covariates in a healthy population was Strati et al., where authors showed age- and gender-associated changes in fungal community, more specifically higher fungal richness in females compared to males, and in adults compared to earlier stages of life [69].

### Associations within and between bacterial and fungal communities

The Pearson correlation test was used to identify associations inside bacterial and fungal sets of OTUs as well as between bacteria and fungi. A total of 41, 77 and 59 associations were found for bacteria vs. bacteria, fungi vs. fungi and bacteria vs. fungi comparisons, respectively (FDR < 0.05) (S2 and S4 Figs).

The bacterial communities were dominated by positive correlations with the only exception of a weak negative correlation between *Blautia* (B_OTU2) and *Prevotella* (B_OTU4). We found no indications of closely related bacterial groups exhibiting stronger correlations and the number of correlations per individual phylum was proportional to its respective relative abundance (S2 Fig).

Associations in the fungal community must be inspected with care because of the bias introduced by low prevalence fungi. Here, strong associations occur as a result of co-occurrence in a small fraction of samples, potentially originating from the same food source. Consequently, we observed significantly more associations in fungal community compared to bacterial. When focusing solely on high prevalence fungi, we identified a negative correlation between *S*. *cerevisiae* (F_OTU1) and both the *C*. *albicans* (F_OTU2) and the unclassified *Debaryomyces* (F_OTU4) to be the most prominent (S3 Fig).

Detected associations between bacteria and fungi span across the top 6 most abundant bacterial phyla (*Firmicutes*, *Bacteroides*, *Actinobactria*, *Proteobacteria*, *Verrucomicrobia* and *Tenericutes*) and top 2 most abundant fungal phyla (*Ascomycota* and *Basidiomycota*). Dominated by positive correlations, they show random distribution and no preference towards any particular taxonomic group (S4 Fig).

## Conclusions

The variability in the healthy human gut microbiota remains largely unexplained despite increasing effort to decipher the microbial patterns with host-specific and environmental factors. In this study we analysed bacterial and fungal communities in a cohort of 186 healthy individuals. Consistent with previous studies [8,9,62], we also report a low per sample fungal diversity, accompanied by a high interindividual variability, which is most likely a consequence of foodborne transient fungi. Out of 13 questionnaire-based host and environmental factors we report 3 significant host covariates. These are age- and gender-associated differences in bacterial communities and the rate of physical activity associated differences in fungal community. Fungal community is known to be largely affected by short-term diet, therefore we assume that the observed patterns highly depend on different diets individuals reported according to the time they dedicated to recreational activities and sport. We identified seven bacterial core genera, out of which three (*Faecalibacterium*, *Bacteroides* and *Roseburia)* commonly appear as core candidates in related studies originating from different geographic regions, including European, American and Mongolian cohorts. The consensus on these core genera, especially in such a variety of studied populations, suggests their pivotal role in the gut, that up to date remains undisclosed.

## Supporting information

S1 Fig Correlation of fungal OTUs with fungal community Shannon diversity. Bar plot shows Pearson correlation coefficient for fungal OTUs that significantly increase (blue) and decrease (red) with fungal community Shannon diversity index.
(TIF)

S2 Fig Associations between bacterial OTUs. Coloured squares on the heat map indicate significant Pearson correlations (false discovery rate (FDR) < 0.05) between bacterial OTUs. Blue shades indicate positive and red shades indicate negative correlation.
(TIF)

S3 Fig Associations between fungal OTUs. Coloured squares on the heat map indicate significant Pearson correlations (false discovery rate (FDR) < 0.05) between fungal OTUs. Blue shades indicate positive and red shades indicate negative correlation.
(TIF)

S4 Fig Associations between bacterial and fungal OTUs. Coloured squares on the heat map indicate significant Pearson correlations (false discovery rate (FDR) < 0.05) between bacterial and fungal OTUs. Blue shades indicate positive and red shades indicate negative correlation.
(TIF)

S1 Table Bacterial OTUs statistics and taxonomy. Table with all bacterial OTUs, percent of samples they appear in, percent of total number of obtained reads they include and their respective taxonomical classification (phylum and the highest taxonomical level to which they reliably classify). Numbers in the parenthesis in the taxonomy column indicate the percent of identity between OTU representative read and the best match in the RDP training set (v.9) reference base. Members of core community are highlighted with grey.
(XLSX)

S2 Table Fungal OTUs statistics and taxonomy. Table with all fungal OTUs, percent of samples they appear in, percent of total number of obtained reads they include and their respective taxonomical classification (phylum and the highest taxonomical level to which they reliably classify). Numbers in the parenthesis in the taxonomy column indicate the percent of identity between OTU representative read and best match in the UNITE reference base. Members of core community are highlighted with grey.
(XLSX)

S1 Appendix Fungal sequence data files for seven samples not available on the Metagenomics RAST (MG-RAST) database server (http://metagenomics.anl.gov/) under the project access number mgp85661 (https://www.mg-rast.org/linkin.cgi?project=mgp85661) and the table with metadata for all samples. (ZIP)
