# Supplementary figures and images for: Different host factors are associated with patterns in bacterial and fungal gut microbiota in Slovenian healthy cohort
Source: PLoS One. 2018 Dec 20;13(12):e0209209. doi: 10.1371/journal.pone.0209209 (PMC6301613; doi:10.1371/journal.pone.0209209)

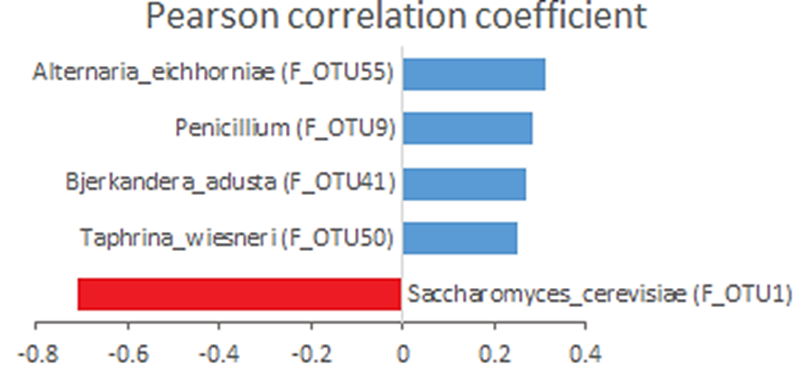

Supplement: S1 Fig — Bar plot shows Pearson correlation coefficient for fungal OTUs that significantly increase (blue) and decrease (red) with fungal community Shannon diversity index. (TIF) [file pone.0209209.s001.tif]

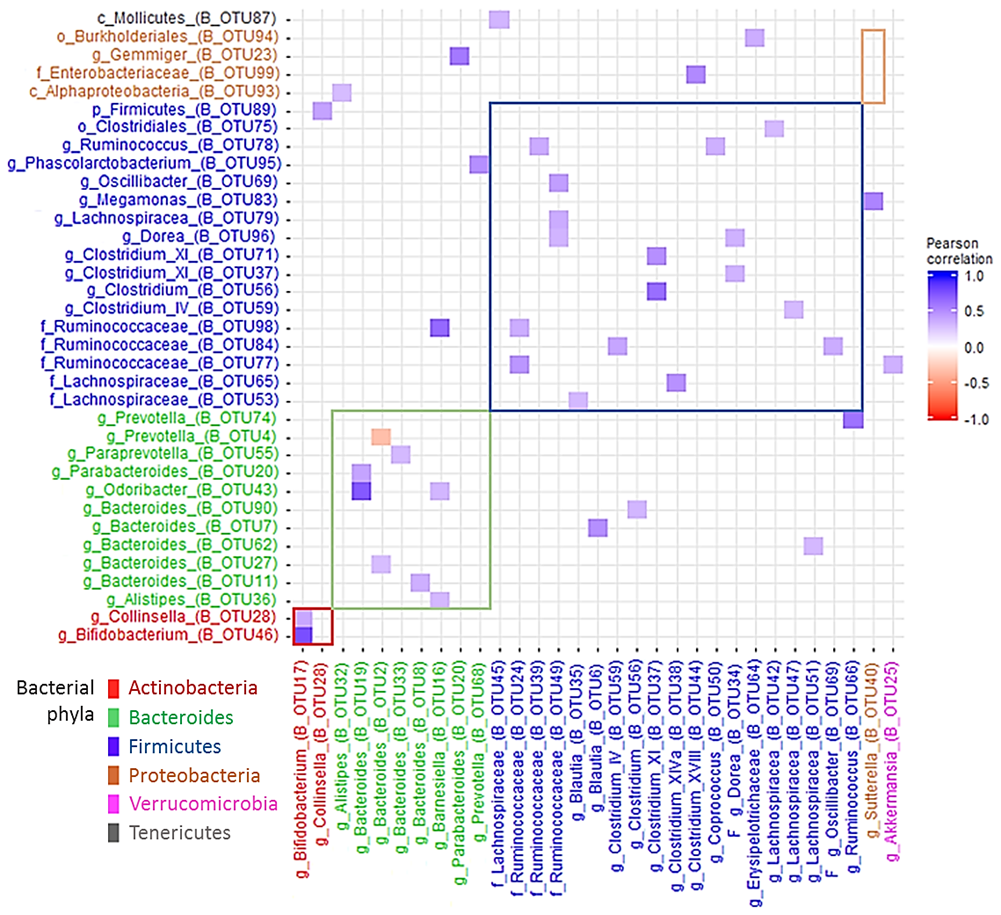

Supplement: S2 Fig — Coloured squares on the heat map indicate significant Pearson correlations (false discovery rate (FDR) < 0.05) between bacterial OTUs. Blue shades indicate positive and red shades indicate negative correlation. (TIF) [file pone.0209209.s002.tif]

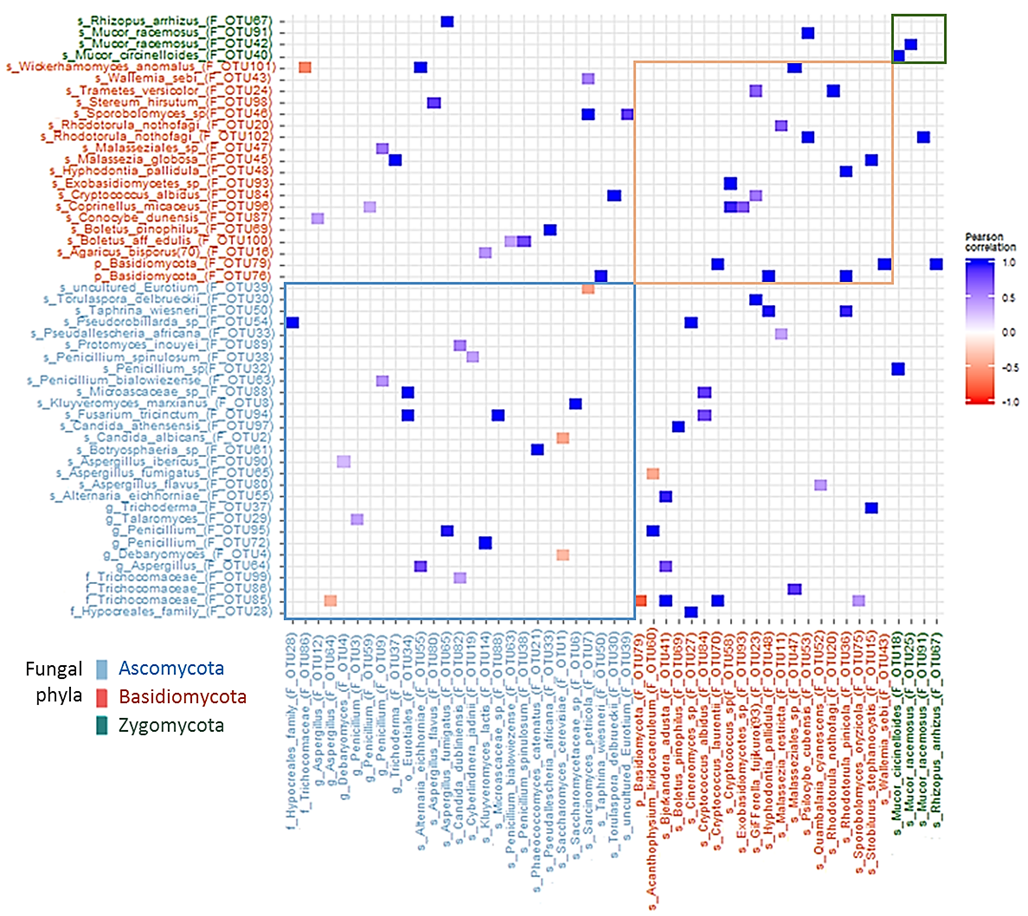

Supplement: S3 Fig — Coloured squares on the heat map indicate significant Pearson correlations (false discovery rate (FDR) < 0.05) between fungal OTUs. Blue shades indicate positive and red shades indicate negative correlation. (TIF) [file pone.0209209.s003.tif]

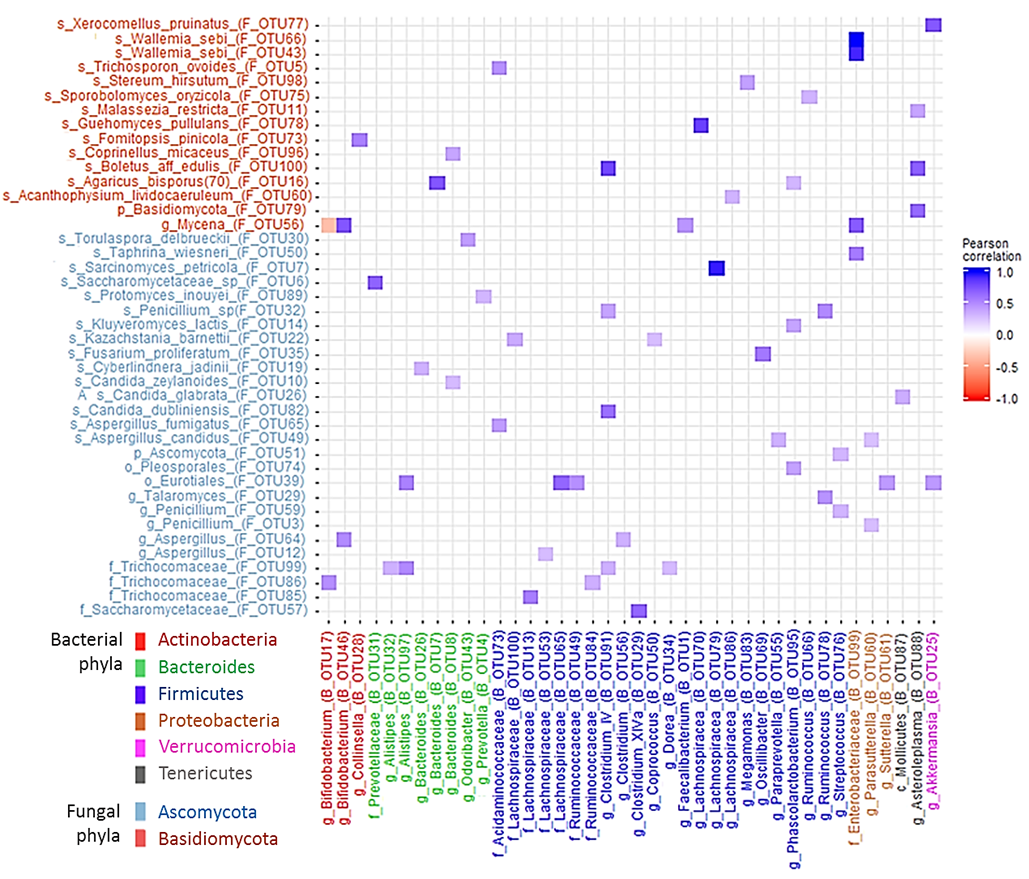

Supplement: S4 Fig — Coloured squares on the heat map indicate significant Pearson correlations (false discovery rate (FDR) < 0.05) between bacterial and fungal OTUs. Blue shades indicate positive and red shades indicate negative correlation. (TIF) [file pone.0209209.s004.tif]
